# Glucagon-like peptide 1 receptor activation regulates cocaine actions and dopamine homeostasis in the lateral septum by decreasing arachidonic acid levels

**DOI:** 10.1038/tp.2016.86

**Published:** 2016-05-17

**Authors:** I A Reddy, J A Pino, P Weikop, N Osses, G Sørensen, T Bering, C Valle, R J Bluett, K Erreger, G Wortwein, J G Reyes, D Graham, G D Stanwood, T A Hackett, S Patel, A Fink-Jensen, G E Torres, A Galli

**Affiliations:** 1 Neuroscience Program, Vanderbilt University School of Medicine, Nashville, TN, USA; 2 Instituto de Química, Facultad de Ciencias, Pontificia Universidad Católica de Valparaíso, Valparaíso, Chile; 3 Department of Pharmacology and Therapeutics, College of Medicine, University of Florida, Gainesville, FL, USA; 4 Laboratory of Neuropsychiatry, Department of Neuroscience and Pharmacology, University of Copenhagen, Copenhagen, Denmark; 5 Psychiatric Centre Copenhagen, University Hospital Copenhagen, Copenhagen, Denmark; 6 Departamento de Ciencias Básicas, Universidad de Viña del Mar, Viña del Mar, Chile; 7 Department of Psychiatry, Vanderbilt University Medical Center, Nashville, TN, USA; 8 Department of Molecular Physiology and Biophysics, Vanderbilt University Medical Center, Nashville, TN, USA; 9 Department of Public Health, University of Copenhagen, Copenhagen, Denmark; 10 Department of Biomedical Sciences and Center for Brain Repair, Florida State University, Tallahassee, FL, USA; 11 Department of Hearing and Speech Sciences, Vanderbilt University Medical Center, Nashville, TN, USA

## Abstract

Agonism of the glucagon-like peptide 1 (GLP-1) receptor (GLP-1R) has been effective at treating aspects of addictive behavior for a number of abused substances, including cocaine. However, the molecular mechanisms and brain circuits underlying the therapeutic effects of GLP-1R signaling on cocaine actions remain elusive. Recent evidence has revealed that endogenous signaling at the GLP-1R within the forebrain lateral septum (LS) acts to reduce cocaine-induced locomotion and cocaine conditioned place preference, both considered dopamine (DA)-associated behaviors. DA terminals project from the ventral tegmental area to the LS and express the DA transporter (DAT). Cocaine acts by altering DA bioavailability by targeting the DAT. Therefore, GLP-1R signaling might exert effects on DAT to account for its regulation of cocaine-induced behaviors. We show that the GLP-1R is highly expressed within the LS. GLP-1, in LS slices, significantly enhances DAT surface expression and DAT function. Exenatide (Ex-4), a long-lasting synthetic analog of GLP-1 abolished cocaine-induced elevation of DA. Interestingly, acute administration of Ex-4 reduces septal expression of the retrograde messenger 2-arachidonylglycerol (2-AG), as well as a product of its presynaptic degradation, arachidonic acid (AA). Notably, AA reduces septal DAT function pointing to AA as a novel regulator of central DA homeostasis. We further show that AA oxidation product γ-ketoaldehyde (γ-KA) forms adducts with the DAT and reduces DAT plasma membrane expression and function. These results support a mechanism in which postsynaptic septal GLP-1R activation regulates 2-AG levels to alter presynaptic DA homeostasis and cocaine actions through AA.

## Introduction

Cocaine addiction is a highly prevalent disorder characterized by continued drug use in spite of negative consequences. Despite the harmful effects of cocaine, the development of pharmacotherapies to treat cocaine addiction has been slow. This is because modulators of addictive behavior are limited and we lack a comprehensive understanding of their mechanisms. Starting with the discovery that glucagon-like peptide 1 (GLP-1) regulates amphetamine-induced locomotion,^1^ GLP-1 receptor (GLP-1R) agonism has been shown to reduce the rewarding properties of cocaine and other drugs of abuse in rodents.^2, 3, 4^

GLP-1 is both a hormone produced by L cells of the intestine and a neuropeptide produced by the nucleus of the tractus solitarius.^5, 6^ Notably, GLP-1 synthetic analogs are already approved to treat type 2 diabetes and obesity in humans.^7, 8^ These synthetic derivatives have a greatly extended half-life over endogenous GLP-1.^5, 7^ As such, they remain in circulation long enough to cross the blood–brain barrier.^9^ The importance of the brain bioavailability of GLP-1 analogs is underscored by the prominent expression of the GLP-1R within the brain.^10^ Ex-4 is one such synthetic GLP-1 analog that has been used extensively to demonstrate that GLP-1R activation reduces the preference and actions of cocaine, amphetamine, alcohol and nicotine.^1, 2, 11, 12, 13, 14, 15, 16, 17^ Importantly, the *GLP-1*R 168Ser allele has been associated with increased measures of alcohol self-administration in humans.^17^ These data suggest that GLP-1R signaling might regulate common mechanisms within the brain's reward system.

The neurotransmitter dopamine (DA) underlies the rewarding properties of drugs of abuse, as well as natural rewards.^18^ It is released at DA terminals during drug intake and in anticipation of the consumption of drugs.^18, 19^ We therefore hypothesized that the effects of GLP-1R stimulation on cocaine reward would occur at DA nodes exhibiting GLP-1R signaling. One such node is found within the forebrain lateral septum (LS). The LS possesses a high concentration of GLP-1Rs and contains DA projections.^10^ In addition, the LS has historically been linked to reward; it was identified by Olds and Milner^20^ as a potent site of electrical self-stimulation in rats.^20^ Since then, several studies have found that the LS is fundamental to the rewarding properties of drugs of abuse.^3, 21, 22, 23^ Most notably, the Aston-Jones group has found that the LS is an important relay center between the hippocampus and the ventral tegmental area (VTA) for information about cocaine–context associations and that the LS is necessary for the formation of cocaine conditioned place preference (CPP). ^22, 23^

Recently, Harasta *et al.*^3^ found that endogenous signaling through the GLP-1R in the LS acts to dampen cocaine-induced locomotion and cocaine CPP, both considered DA-associated behaviors.^3^ In the current study, we sought to determine the mechanism by which GLP-1R stimulation modulates septal DA homeostasis and the actions of cocaine. Cocaine blocks DA reuptake via the DA transporter (DAT), resulting in high levels of synaptic DA. Here we demonstrate that GLP-1R stimulation inhibits the ability of cocaine to increase extracellular DA levels. This phenomenon is associated with increased DAT surface expression and function mediated by a reduction in endocannabinoid and arachidonic acid (AA) levels in the LS. These findings reveal a novel mechanism of GLP-1R action in brain and provide a more comprehensive understanding of how emerging GLP-1R-targeted therapies affect signaling in the brain.

## Materials and methods

### Animals

Male NMRI mice (Taconic, Lille Skensved, Denmark) were used for microdialysis and c-fos experiments.^4^ Sprague Dawley rats, weighing 320–345 g, were also used for the study (Taconic). All other *in vivo* experiments used adult male C57BL/6 mice from Jackson Laboratories or bred internally at the Vanderbilt University. All the animals were kept at room temperature in a 12 h light/dark cycle with free access to food and water. All the experiments were performed during the light cycle. For immunohistochemical characterization of GLP-1R localization, bacterial artificial chromosome transgenic mice containing a fluorescent reporter expressed in GLP-1R-expressing cells were generated as described in Supplementary Information. All the experiments were in accordance with directives of ‘Principles of Laboratory Animal Care' (NIH publication No. 85–23) and approved by either the Danish Experimental Animal Inspectorate and the council of the European Communities or the Vanderbilt Institutional Animal Care and Use Committee.

### Drugs and materials

The suppliers for all chemicals not otherwise identified can be found in the Supplementary Information.

### Immunohistochemistry for DAT and mApple

The brains from GLP-1R bacterial artificial chromosome transgenic mice were extracted, post-fixed overnight in PFA and sectioned by vibratome (75 μm). The sections were immunostained and imaged as described in the Supplementary Information.

### High-speed chronoamperometry

The slices containing LS (300 μm) were prepared and chronoamperometry performed as previously described.^24^ High KCl artificial cerebrospinal fluid (with 30 mm KCl, 97.5 mm NaCl) was applied to the bath for 2 min to induce DA release. The oxidative signal measured in the slices is attributable to DA as the reduction/oxidation charge ratio is in the range 0.6–1.0.

### c-fos expression

The procedure for c-fos IHC is as previously described.^25^ Briefly, the coronal brain sections (15 μm) were cut through septum using a cryostat. Synthetic oligonucleotide DNA probes (DNATechnology, Aarhus, Denmark) were used for the visualization of c-fos mRNA by autoradiography. The probe sequence was  5′-CGGGCAGTGGCACGTCTGGATGCCGGCTGCCTTGCCTTCTCTGACTGC-3′.

### *In vivo* microdialysis in mice

Microdialysis was performed as described previously^4^ with few modifications. The dialysis probe was positioned in LS, anteroposterior: 0.5 mm, mediolateral: 0.3 mm relative to the bregma and dorsoventral: −2.2 mm relative to skull surface.^26^ The microdialysis probe was perfused at a rate of 0.9 μl min^−1^ with artificial cerebrospinal fluid. The first two 30-min samples were discarded to obtain stable basal values. Hereafter, two 30-min fractions were collected to establish baseline DA levels. Subsequently, the mice were injected with Ex-4 or vehicle, and three 30-min fractions were collected. The mice were then subjected to local perfusion with cocaine (50 μm) for 30 min and five 30-min fractions were collected. All the fractions were assayed immediately after collection using high-performance liquid chromatography with electrochemical detection.^27^

### *Ex vivo* slice protein biotinylation assay

The procedure for biotinylation of live brain slices has been validated and was performed at 28 °C as described previously.^28^

### *Ex vivo* slice [3H]DA transport assay

The slices were obtained as described above and allowed to recover (⩾1 h at 28 °C) with continuous oxygenation. Following the recovery, the slices were treated with the drug (that is, GLP-1) for 10 min followed by the application of 50 nm [^3^H]DA for 10 min with the drug still present at 28 °C. When Ex-9 was used, the slices were pretreated with Ex-9 for 10 min before the addition of drug/vehicle. Total protein was taken, and the samples were processed for protein concentration using a Bio-Rad (Philadelphia, PA, USA) protein assay and spectrometry at 595 nm. Equivalent volumes of the sample were added to 3 ml of scintillation fluid (Ecoscint H, National Diagnostics, Atlanta, GA, USA) and radioactive counts were measured by scintillation counter. [^3^H]DA was divided by protein and normalized to average control uptake from paired vehicle-treated slices from the same animal. The uptake protocol and DAT specificity were validated in slices treated with DAT blocker GBR-12909 (Sigma Aldrich, St. Louis, MO, USA), which blocked uptake in LS by 58.2% (*t*(5)=11.97; *P*<0.0001 by Student's *t*-test; *n*=6).

### 2-AG and AA measurements

The mice were injected with Ex-4 or vehicle (intraperitoneally) 30 min before being killed. The mice were rapidly decapitated and the brains were obtained and blocked to obtain DS and LS punches. Punches were quickly placed into Eppendorf tubes on dry ice and stored at −80 °C until use. The samples were homogenized in methanol and centrifuged; water was added to the supernatant for a final ratio of 70:30 methanol:water. Liquid chromatography/mass spectrometry was performed as previously described.^29^ Following liquid chromatography/mass spectrometry, excess methanol was suctioned off the pellets. The pellets were resuspended and processed for protein concentration using a Bio-Rad protein assay and spectrometry at 595 nm.

### Cell culture

Rat pheochromocytoma cells (PC12) were obtained from the American Type Culture Collection (Manassas, VA, USA), and were cultured in Dulbecco's Modified Eagle's medium supplemented with 6% fetal bovine serum (heat inactivated), 6% horse serum (heat inactivated), 1 mm glutamine, 100U ml^−1^ penicillin and 100 μg ml^−1^ streptomycin at 37 °C in a humidified 5% CO_2_ incubator.^30^ The PC12 cells overexpressing human DAT fused to GFP (hDAT cells) were stably transfected with the plasmid peGFP-C2-hDAT using Lipofectamine 2000 and were cultured under the same conditions. Krebs–Henseleit buffer was used (144 mm NaCl, 4.2 mm KCl, 1.6 mm MgCl_2_, 1.6 mm KH_2_PO_4_, 10 mm HEPES, pH 7.4) and Fe^+3^ (197 μm)/ascorbate (19.7 mm) solutions were prepared in Krebs–Henseleit buffer. In some cases, salicylamine, a γ-KA scavenger, was added to the culture media and preincubated with the cells for 1 h at 37 °C before further treatments. The cell viability was measured using Trypan blue staining.

### PC12 transport assays

The DAT-mediated uptake of DA in PC12 cells overexpressing a GFP-tagged DAT (hDAT) has been described previously.^31^

### Cell surface biotinylation in hDAT cells

The cell surface biotinylation was performed as described by Egaña *et al.*^31^ with some modifications.

### Immunoprecipitations

Immunoprecipitations were performed using 1 mg total protein. To immunoprecipitate DAT-GFP, the cell homogenates were incubated overnight at 4 °C with a monoclonal anti-GFP antibody (G-6539, 1:100), followed by the addition of 50 μl of a mixture of protein A and protein G Sepharose beads (2 h at 4 °C in rotatory shaker). The immunoprecipitated proteins were recovered by centrifugation at 14 000 *g* for 2 min (4 °C), washed with ice-cold buffer D, and resuspended in protein sample buffer containing βME. The proteins were separated by SDS-polyacrylamide gel electrophoresis and transferred to PVDF membranes before incubation with an antibody against γ-KA-lysil adduct (neuroketals antibody, C-17, 1:400) and anti-DAT (MAB369, 1:1000).

### Analyses

The data are presented as means±s.e.m. The data were examined with Student's *t*-test for comparisons of only two data sets and one-way or two-way analysis of variance for comparisons of multiple groups, followed by Tukey's- or Bonferroni-corrected comparisons. In *ex vivo* [^3^H]DA uptake experiments, samples with low protein content (<6 μg ml^−1^) were excluded from the analysis (*n*=1). Optical density of bands was determined using Image J software. The statistical analysis was performed with GraphPad Prism software, version 5.02 (GraphPad Software, San Diego, CA, USA). A *P*-value <0.05 defined the statistical significance.

The methods for Supplementary Figures can be found in the Supplementary Information.

## Results

### GLP-1R-expressing neurons are juxtaposed to DA terminals in the LS

**Figure 1:**
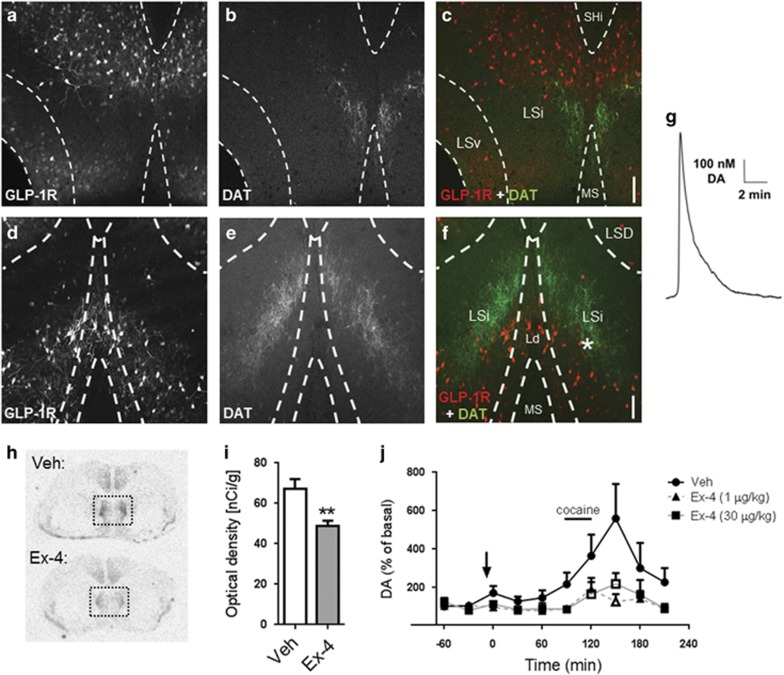
The glucagon-like peptide 1 receptor (GLP-1R) is highly expressed in the lateral septum (LS) where GLP-1R agonists block cocaine-induced dopamine (DA) *in vivo.* (**a-c**) Cells expressing the GLP-1R (white in **a**, red in **c**) and terminals expressing dopamine transporter (DAT; white in **b**, green in **c**) in rostral LS (GLP-1R in **a**, DAT in **b**, merge in **c**). Confocal images in z stack through 13 planes taken with a × 10 objective. (**d-f**) Cells expressing the GLP-1R (white in **d**, red in **f**) and terminals expressing DAT (white in **e**, green in **f**) in caudal LS (GLP-1R in **d**, DAT in **e**, merge in **f**). Confocal images in z stack through 14 planes taken with a × 10 objective. (**g**) DA release induced by 30 mm KCl (representative of *n*=3) in section from the caudal LS. Approximate site of recording is demarcated by * in **f**. Ld, laterodorsal tegmental nucleus; LSd, dorsal LS; LSi, intermediate LS; LSv, ventral LS; MS, medial septum; SHi, septohippocampal nucleus. Dotted lines delineate region boundaries.^26^ Scale bars, 100 μm. (**h**) Representative c-fos expression in the LS 30 min following vehicle (top) or Ex-4 (30 μg kg^−1^, intraperitoneally, bottom) injection. Dotted box includes the LS. (**i**) Quantification of optical density analysis of c-fos autoradiographs in LS (*t*(14)=3.26; **P<0.01 by Student's *t*-test; *n*=8). (**j**) Cocaine (50 μm) greatly increased septal DA levels in mice pretreated with saline (intraperitoneally, solid circles). This cocaine-induced DA increase is significantly diminished in mice pretreated with Ex-4 (1 μg kg^−1^ intraperitoneally, solid triangles; 30 μg kg^−1^ intraperitoneally, solid squares). Extracellular concentrations of DA are expressed as the percentage of basal levels in two fractions collected before the intervention; significant analysis of variance. Open symbols indicate significance by *post hoc* test where *P*<0.001 at 150 min and *P*<0.05 at 120 min; *n*=5–6 per group.

The GLP-1R is highly expressed within the LS,^10^ a brain region in which DAT-expressing DA terminals reside.^32^ Here we first describe their proximity and anatomical distribution in this region (Figure 1). To this end, we generated a transgenic mouse expressing a bacterial artificial chromosome for the protein mApple under the control of the promoter for the GLP-1R. The mApple signal was amplified by immunostaining (Figures 1a, c, d and f) and DAT was labeled in parallel (Figures 1b, c, e and f). Within the rostral LS (Figures 1a–c), the DA terminals are found medially in the intermediate LS subregion (LSi), directly adjacent to GLP-1R-expressing cells found mostly in the dorsal LSi. We observed visible overlap between these cell bodies/projections and DA terminals. Within the caudal LS (Figures 1d–f), there is similar evidence for this anatomical overlap. The DA terminals are labeled as two strips extending dorsomedially to ventrolaterally in the LSi. The GLP-1R-expressing cells are also found in the caudal LSi, but the cell bodies additionally extend further into the more medial laterodorsal tegmental area. Importantly, we show that DA terminals within this region release DA: superfusing LS slices with an artificial cerebrospinal fluid solution containing high [K^+^] causes DA release as recorded by high-speed chronoamperometry (Figure 1g; approximate electrode placement is denoted by * in Figure 1f). The use of mApple for identifying GLP-1R-expressing neurons was validated by *in situ* hybridization (Supplementary Figure 1).

### Systemic administration of a GLP-1R agonist reduces cocaine-induced DA elevation and c-fos expression in the LS

We next tested whether a therapeutically relevant administration (that is, systemic administration) of Ex-4 was capable of altering neuronal activity in the LS. We first evaluated the effect of Ex-4 on the activation of septal neurons by measuring c-fos expression. Ex-4 administration (intraperitoneally) 30 min before killing decreased basal c-fos gene expression by *in situ* hybridization relative to vehicle-treated mice (Figures 1h and i). This result indicates that systemic administration of Ex-4 alters neuronal activity in the LS.

The psychogenic and addictive properties of cocaine are mediated, at least in part, through the blockade of DAT-mediated DA reuptake and an increase in extracellular DA.^33, 34^ Therefore, signaling pathways able to modulate or diminish this DA increase offer enormous therapeutic potential. We determined the effect of systemic Ex-4 on local cocaine-induced changes in DA homeostasis *in vivo* within the LS, as measured by microdialysis in freely moving mice. The perfusion of cocaine through the microdialysis probe increased septal DA ~5-fold (Figure 1j; F(9,49)=2.73, *P*=0.014 by analysis of variance). Systemic pretreatment with Ex-4 (intraperitoneally) 90 min prior impaired the cocaine's ability to significantly increase extracellular DA. These results were paralleled in rats (Supplementary Figure 2A), demonstrating across species that GLP-1R activation diminishes the cocaine's ability to increase extracellular DA in the LS.

### Local GLP-1R signaling blocks cocaine-induced elevation in DA, promotes septal DAT membrane expression and increases DA uptake

We next sought to determine whether local GLP-1R activation could account for observed systemic effects of Ex-4 on the LS. Ex-4 or vehicle was perfused through the microdialysis probe concurrently with cocaine (Supplementary Figure 2B). The local administration of Ex-4 impaired the ability of cocaine to increase extracellular DA levels. This finding further highlights the role of septal GLP-1Rs in modulating cocaine's ability to enhance DA function.

**Figure 2:**
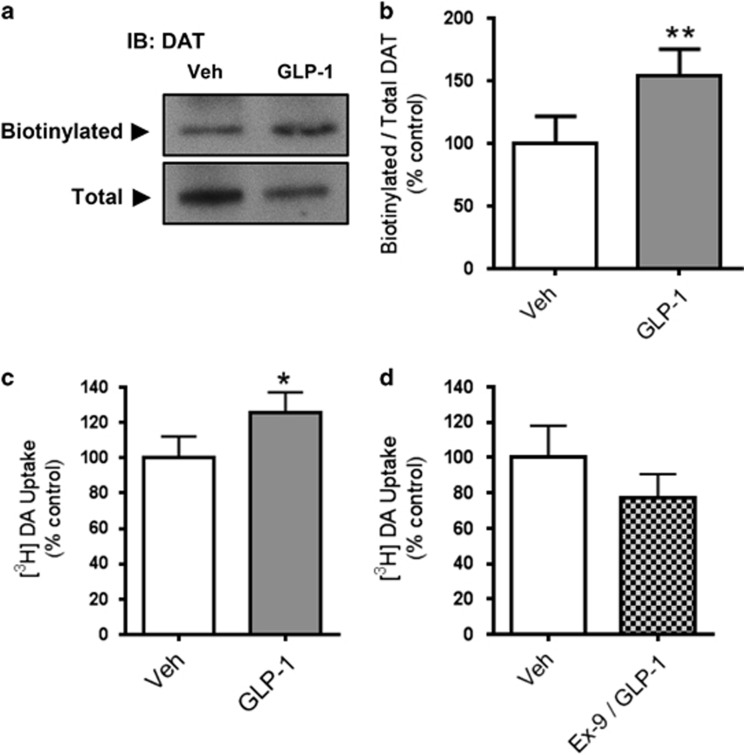
Local activation of the glucagon-like peptide 1 receptor (GLP-1R) promotes septal dopamine transporter (DAT) membrane expression and [^3^H]DA uptake. (**a**) Representative immunoblot of surface and total DAT following slice treatment with vehicle (artificial cerebrospinal fluid, aCSF) or 1 nm GLP-1. (**b**) Ratio of DAT surface expression in GLP-1-treated *ex vivo* septal slices as percentage of average vehicle values (*t*(3)=10.12; **P<0.01 by Student's *t*-test; *n*=4). The slices were treated with 1 nm GLP-1 for 20 min. (**c**) [^3^H]DA uptake in the LS from *ex vivo* slice preparation following 20 min treatment with vehicle (aCSF) or 10 nm GLP-1. Uptake was significantly elevated (*t*(4)=2.35; *P<0.05 by Student's *t*-test; *n*=5). (**d**) Pretreatment with 100 nm Ex-9 for 10 min blocks the increase in [^3^H]DA uptake following treatment with 10 nm GLP-1 (*t*(3)=1.047; *P*=0.19 by Student's *t*-test; *n*=4).

Multiple mechanisms could explain how local GLP-1R signaling impairs cocaine-induced increases in extracellular DA in the LS. However, DAT-mediated DA reuptake is the primary mechanism regulating extracellular DA levels,^35^ a process dynamically regulated by DAT plasma membrane expression.^36, 37, 38, 39, 40^ Notably, DAT expression is inversely proportional to the ability of cocaine to cause its behavioral effects.^41, 42^ Therefore, we hypothesized that GLP-1R signaling regulates DA homeostasis in the LS, as well as cocaine's ability to increase extracellular DA, by controlling DAT surface expression. In support of this mechanism, GLP-1 application increased surface levels of DAT as measured by LS slice biotinylation^43^ (Figures 2a and b). To determine whether the elevation of DAT surface expression translated to functional changes in DAT activity, we measured uptake of [^3^H]DA in septal slices treated with GLP-1. GLP-1 significantly increased DA uptake (Figure 2c). This increase was specific to GLP-1R signaling, as demonstrated by its blockade by the selective GLP-1R antagonist, exendin-(9–39)-amide (Ex-9; Figure 2d).^44^ Ex-9 alone had no significant effect (91%±13% *t*(3)=0.6749; *P*=0.55 by Student's *t*-test; *n*=4). These studies were performed in slices in which only the local circuits are intact; therefore, we can assume that our observations were the result of local GLP-1R stimulation and not the result of feed-forward circuit-level changes. Together, these data demonstrate that GLP-1R signaling increases both DAT expression at the plasma membrane and DA uptake, suggesting that GLP-1R signaling might regulate cocaine actions in the LS through DAT-dependent mechanisms.

### Ex-4 reduces levels of the retrograde messenger, 2-AG, and lowers levels of AA in the LS

**Figure 3:**
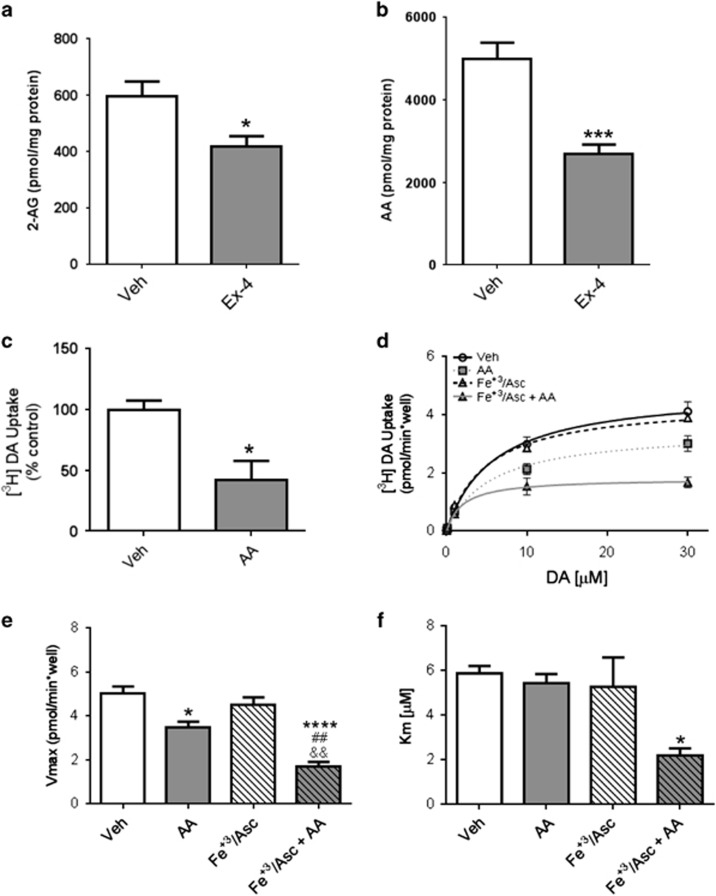
Treatment of mice with Ex-4 reduces septal arachidonic acid (AA) levels and AA is a strong modulator of dopamine transporter (DAT) function in lateral septum (LS). (**a**) Ex-4 (2.4 μg kg^−1^, intraperitoneally) administered *in vivo* 30 min before killing resulted in lower levels of septal 2-AG (*t*(11)=2.62; *P<0.05 by Student's *t*-test; *n*=6–7). (**b**) Exenatide (Ex-4; 2.4 μg kg^−1^, intraperitoneally) administered 30 min before sacrifice reduces levels of septal AA (*t*(10)=5.018; ***P<0.001 by Student's *t*-test; *n*=6). (**c**) Eighty micromolar AA applied for 20 min to slices containing the LS reduces [^3^H]DA uptake to 42% of vehicle (dimethyl sulfoxide)-treated slices (*t*(3)=2.59; *P<0.05 by Student's *t*-test; *n*=4). (**d**) [^3^H]DA uptake kinetics in human DAT (hDAT) cells treated either with vehicle (open circles) or Fe^+3^/Asc (open triangles) or with 40 μm AA for 1 h at 37 °C in absence (gray squares) or presence of Fe^+3^/Asc (gray triangles). The kinetic parameters (**e**) Vmax and (**f**) Km were obtained from the Michaelis–Menten fit to the influx of [^3^H]DA. Each value represents mean±s.e.m. of ⩾ three independent experiments. *P<0.05, ****P<0.001, ^##^*P*<0.01 and ^&&^*P*<0.01 indicate significant differences between vehicle (Veh) and AA, Veh and Fe^+3^/Asc+AA, AA and Fe^+3^/Asc+AA and Fe^+3^/Asc and Fe^+3^/Asc+AA, respectively. Significant analysis of variance followed by *post hoc* tests.

Our findings thus far suggest that septal GLP-1R agonism affects DAT expression and function locally. However, the GLP-1R is predominantly expressed postsynaptically (Figure 1 and Supplementary Figure 1), whereas the DAT is located on presynaptic DA terminals (Figure 1). This raised the possibility that GLP-1R signaling regulates DAT function through a retrograde signal. 2-AG is a well-established endogenous cannabinoid retrograde messenger whose signaling has been shown to modify reward and feeding.^45, 46, 47, 48^ Of note, cannabinoid CB1 receptor antagonists, which decrease 2-AG signaling, have been shown to decrease cocaine reward.^49^ 2-AG is synthesized postsynaptically, but degraded into free AA in presynaptic axon terminals. Indeed, 2-AG hydrolysis via monoacylglycerol lipase is the primary mechanism for free AA generation in the central nervous system.^50^ Importantly, AA impairs DAT-mediated DA uptake.^51, 52, 53^ Thus we sought to determine whether GLP-1R activation modulates 2-AG-AA signaling. To this end, mice were injected with Ex-4 (intraperitoneally) 30 min prior to sacrifice, and the LS was punched and analyzed by mass spectrometry for 2-AG and free AA levels.^50^ We found that Ex-4 significantly reduced both 2-AG and AA levels in the LS (Figures 3a and b), pointing towards reduced endocannabinoid metabolism in the LS. This phenomenon was not observed in the dorsal striatum (DS), where DAT is expressed in high quantities but GLP-1R-expressing cells are only sparsely distributed (Supplementary Figure 3). These data demonstrate region-specific regulation by GLP-1R signaling of 2-AG levels and its metabolism.

### AA reduces DA uptake and DAT surface expression

Among its functions as a signaling molecule, AA has been previously shown to impair DAT-mediated DA uptake.^51, 52, 53^ Consistent with findings from heterologous cell lines and striatal synaptosomes,^51, 52, 53^ we first demonstrated that AA application to LS slices significantly reduced DA uptake (Figure 3c).

Although we have established that AA inhibits DAT function in the LS, the mechanism(s) responsible for this effect are unknown. To define these molecular mechanisms, we adopted PC12 cells stably transfected with the human DAT (hDAT cells). The PC12 cells contain vesicles capable of neurotransmitter release.^54^ These vesicles hold catecholamines (mostly DA) and as such they were suitable for our molecular studies. The incubation of hDAT cells with AA alone decreased the DAT maximal velocity (Vmax) to 69% of control condition (Figures 3d and e). Notably, psychostimulants are known to induce oxidative stress,^55, 56, 57^ whereas GLP-1R signaling, which reduces AA levels, is known to reduce it.^58^ Thus, we further explored mechanistically the interplay between oxidative stress and AA levels in terms of DAT function. Here, we treated hDAT cells with AA along with an oxidizing solution containing Fe^+3^ (197 μm) plus ascorbate (19.7 mm, Fe^+3^/Asc; Figures 3d–f). Importantly, Fe^+3^/Asc induces the lipid peroxidation of AA, a process that produces reactive species capable of modifying protein structure and function.^59, 60, 61, 62^ Notably, DAT is highly susceptible to oxidant injury.^63, 64, 65^ We thus posited that AA might exhibit greater inhibitory effects under oxidative conditions. Indeed, a greater decrease in Vmax to 34% of control was obtained when AA was applied in the presence of Fe^+3^/Asc (Figures 3d and e; See also Supplementary Figure 4 for validation of oxidizing conditions and cell viability). However, when hDAT cells were incubated with Fe^+3^/Asc alone, there were no significant changes in DAT activity (Figures 3d and e). These results show that in the presence of AA, there is a significant reduction in DAT function under both oxidizing and non-oxidizing conditions. Notably, a greater loss of function was observed under oxidizing conditions, which also affects DAT's apparent affinity for DA (Figure 3f).

**Figure 4:**
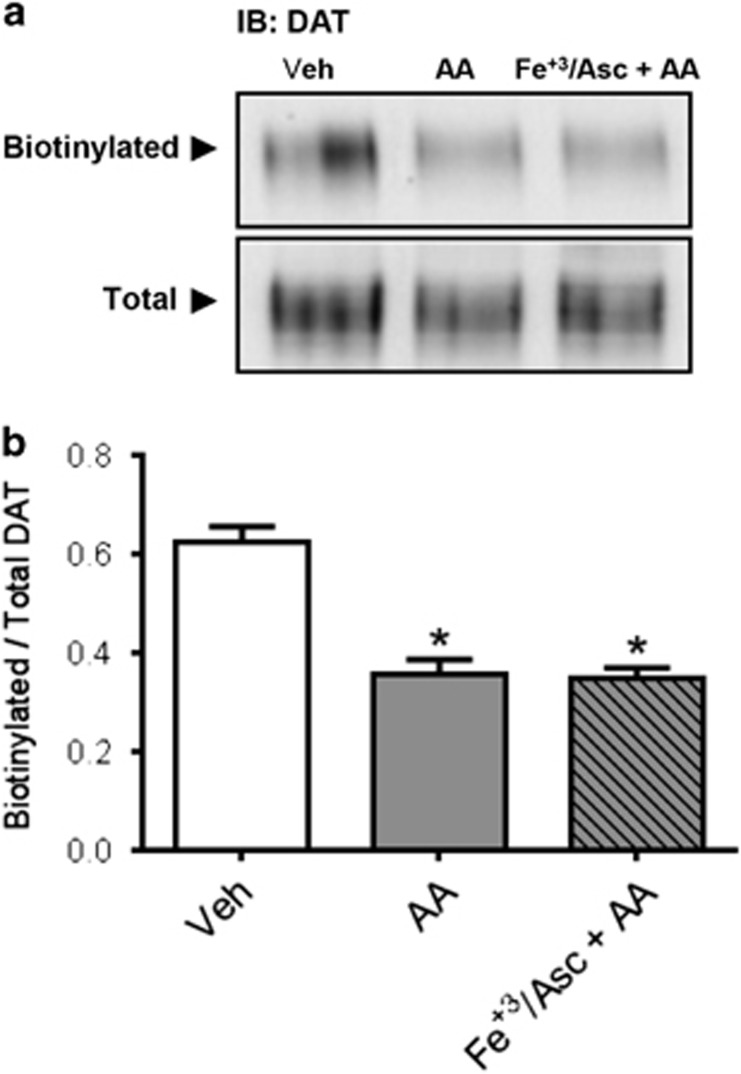
Arachidonic acid (AA) decreases dopamine transporter (DAT) cell surface expression in human DAT (hDAT) cells. (**a**) Cell surface biotinylation and immunoblots for DAT using anti-DAT in extracts from hDAT cells treated with AA (40 μm) for 1 h at 37 °C in the absence or presence of Fe^+3^/Asc. (**b**) Results expressed as the ratio of optical density between biotinylated (surface) and total DAT signals for each experimental condition. Each value represents mean ±s.e.m. of ⩾ three independent experiments. *P<0.05 indicates significant differences with respect to vehicle condition (0.1% ethanol in Krebs–Henseleit buffer). Significant analysis of variance followed by *post hoc* tests.

DAT plasma membrane trafficking is a key regulator of DA transport capacity.^66, 67^ Therefore, the effect of AA on DAT function could be explained by the changes in the cell surface expression. Our results indicate that in hDAT cells, AA, under both oxidative and non-oxidative conditions, decreases significantly and to the same extent plasma membrane DAT (Figures 4a and b) as determined by biotinylation. Therefore, the decrease in DAT function induced by AA alone parallels the decreased transport capacity induced by AA (Figure 3). These data also point to additional mechanisms promoted in terms of DAT function by oxidative stress.

### AA oxidative product, γ-KA, forms a complex with DAT and regulates its function

The oxidation of AA (mediated by free radicals and other reactive oxygen/nitrogen species) yields a series of prostaglandin H_2_ isomers that rearrange to their corresponding γ-ketoaldehydes (γ-KAs), also named isoketals. These γ-KAs react rapidly with lysine residues forming stable adducts, which can modulate the activity of proteins.^59, 60, 61, 62^ At the plasma membrane, DAT localizes to microdomains including lipid rafts. Lipid rafts are regions highly enriched in phospholipids containing AA, and DAT localization to lipid rafts has been associated with regulation of DAT function and trafficking.^68, 69, 70^ Therefore, we determined whether posttranslational modifications of DAT by γ-KAs are associated with changes in DAT function.

**Figure 5:**
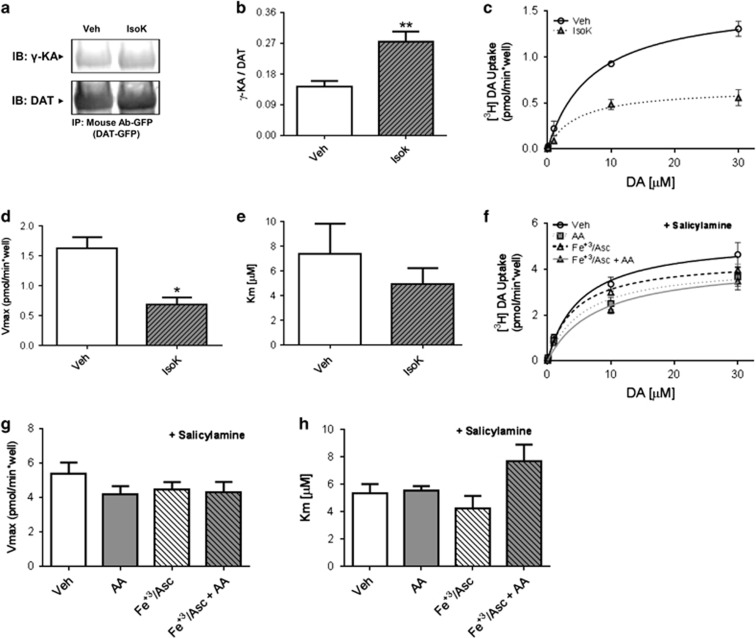
Synthetic isoketals form adducts with human dopamine transporter (hDAT) and decrease its function. (**a**) Immunoblots with anti-γ-KA protein adduct and anti-DAT antibodies using green fluorescent protein (GFP)-DAT immunoprecipitates from hDAT cells treated with synthetic isoketals (1 μm) for 1 h at 37 °C. (**b**) Quantification of immunoprecipitates as a ratio of γ-KA:DAT. Each value represents mean±s.e.m. of ⩾ four independent experiments (*t*(6)=3.94; **P<0.01 by Student's *t*-test; *n*=4). (**c**) Uptake of [^3^H]DA in hDAT cells treated with vehicle (open circles) or with 1 μm synthetic isoketals (gray triangles) for 1 h at 37 °C. The kinetic parameters (**d**) Vmax and (**e**) Km were obtained from the Michaelis–Menten fit to [^3^H]DA uptake. Each value represents mean±s.e.m. of three independent experiments (Vmax: *t*(4)=4.21; *P<0.05 by Student's *t*-test; *n*=3; Km: *t*(4)=0.88; *P=0.43 by Student's *t*-test). (**f**) Influx of [^3^H]DA in hDAT cells pretreated with salicylamine (0.5 mm) and then treated with vehicle (open circles, 0.1% ethanol in Krebs–Henseleit buffer), Fe^+3^/Asc (open triangles) or with 40 μm AA for 1 h at 37 °C in the absence (gray squares) or presence of Fe^+3^/Asc (gray triangles). The kinetic parameters (**g**) Vmax and (**h**) Km were obtained from the Michaelis–Menten fit to the influx of [^3^H]DA. Each value represents mean±s.e.m. of ⩾ three independent experiments. No significant differences were observed between the groups. The statistical analysis was performed with analysis of variance. AA, arachidonic acid; Veh, vehicle.

To determine whether DAT is a target of AA oxidative products (for example, γ-KAs) and whether this interaction is relevant for its function, we conducted experiments using synthetic isoketals (IsoK). The hDAT cells were incubated with IsoK or vehicle for 1 h at 37 °C and cell extracts were used for immunoprecipitation experiments. We observed a significant increase (78%) in the amount of isoketal adducts that co-immunoprecipitated with DAT compared with the control condition (Figures 5a and b). Importantly, incubation with IsoK resulted in a 59% decrease in function compared with vehicle control (Figures 5c and d), with no change in Km (Figure 5e). Furthermore, we evaluated the effects of salicylamine, a selective scavenger of γ-KA, on DA uptake (Figure 5f). In the presence of salicylamine, there were no significant changes in Vmax (Figure 5g) or Km (Figure 5h) when cells were treated with AA under non-oxidative or oxidative conditions with respect to the control condition. This is in contrast to our earlier results in the absence of salicylamine (Figures 3d and e), suggesting that the ability to form γ-KA-DAT adducts is necessary for AA to impair DAT function. Importantly, our findings demonstrate that DAT is a target of AA oxidative products, and that this interaction has profound functional implications.

## Discussion

Since the discovery that the LS is a potent site of electrical self-stimulation in both rodents and humans,^20, 71^ it has historically received relatively little attention as a reward center. Recent studies, however, suggest that it integrates output from traditional reward areas including the VTA and lateral hypothalamus.^22, 23^ In this context, the LS has been described as a relay node from the hippocampus (area CA3) to the VTA, and has been suggested to have a pivotal role in encoding context–reward associations for cocaine.^22^ Notably, functional inactivation of the rostral LS abolished cocaine CPP.^23^ These reports and others^21, 72, 73^ characterize the LS as a brain region critically involved in coordinating the activity of multiple reward centers and reinforce its importance in cocaine reward.

GLP-1Rs are highly expressed in the LS and their signaling has been shown to attenuate the locomotor response to the psychostimulant d-amphetamine,^1^ to reduce cocaine CPP,^14, 16^ and to block self-administration for cocaine in rodents.^4^ Therefore it is conceivable that their signaling within the LS may modulate cocaine CPP and reward. To support this hypothesis, a recent study shows that GLP-1R expression in the LS controls cocaine CPP and locomotion.^3^ However, the mechanism by which GLP-1R signaling fine tunes DA homeostasis and cocaine reward in the LS remains elusive.

Cocaine, by targeting the DAT, increases extracellular DA levels, which leads to cocaine's behavioral actions. DAT expression level is an important factor in determining the effects of cocaine, likely through effects on basal DA tone. For example, extended access to cocaine, which results in escalating cocaine intake, causes lower expression of striatal DAT and reduced DA clearance in rats.^74, 75, 76, 77, 78^ The involvement of DAT expression in cocaine's behavioral actions is further supported by the finding that hyperlocomotor responsiveness to cocaine is significantly enhanced in two different mouse models expressing low levels of DAT,^41, 42^ likely resulting from reduced basal DAT-mediated DA uptake.^41^ In addition, experiments from heterologous cell lines show that cocaine is more potent in inhibiting DA uptake in cells expressing lower amounts of DAT.^79^ Notably, a high percentage of striatal DAT (at least 47%) needs to be occupied by cocaine in addicts before they report subjective effects of the drug,^80^ implying that modulating DAT surface expression alters the reinforcing effects of cocaine in humans. However, it is clear to us that other mechanisms in addition to DAT occupancy participate in the behavioral actions of cocaine. These include altered firing activity of dopaminergic neurons and/or other mechanisms that modify DA release. These kinds of mechanisms have been defined for other drugs of abuse, such as morphine, and may be contributing in parallel to altered cocaine reward.^81^

In this study, we hypothesized that GLP-1R signaling controls, at least in part, cocaine actions by regulating DAT expression/function in the LS. We find that GLP-1R agonism enhances membrane expression of DAT. This increase translates into an increase in transport capacity, and is paralleled by reduced ability of cocaine to increase extracellular DA levels. Our data suggest that increasing the amount of DAT expressed at the cell surface may reduce the extent to which cocaine is effective at increasing extracellular DA within the LS. These data offer an additional mechanism for GLP-1R signaling regulation of the reinforcing and rewarding properties of cocaine without precluding the possibility that GLP-1R signaling may also affect DA release and firing properties of VTA neurons.

Here we find that stimulating the GLP-1R through administration of Ex-4 in a therapeutically relevant manner (systemic administration) reduces septal 2-AG levels. One possibility is that the decrease in 2-AG is due to GLP-1R-mediated stimulation of PI3K,^82^ or inhibition of PTEN,^83^ which will decrease the levels of plasma membrane PIP_2_, one of the precursors for 2-AG synthesis.^84^ We show that in the LS GLP-1R is located postsynaptically. Indeed, 2-AG diffuses across synapses. Therefore, we believe that this loss of 2-AG is causal for the observed decrease in LS AA content as AA is primarily produced in the brain through the presynaptic conversion from 2-AG.^50^ This reduction is important as AA has been shown to inhibit DA uptake in *ex vivo* and *in vitro* preparations,^51, 52, 53^ and that psychostimulants such as amphetamine cause release of AA by activation of PLA_2_.^85^ Consistent with AA-mediated inhibition of septal DA uptake, we show that GLP-1R-mediated reduction in AA signaling is associated with an increase in DA uptake in LS slices. This increase in DAT function stems at least in part from augmented DAT surface expression, pointing to DAT trafficking as the underlying mechanism by which GLP-1R signaling and AA levels controls DA homeostasis in the LS, as depicted by our schematic model (Supplementary Figure 5).

Of note, AA is known to facilitate a pro-inflammatory state in the brain;^86^ GLP-1 analogs, on the other hand, possess both anti-inflammatory and neuroprotective effects.^58, 87, 88^ GLP-1R stimulation has demonstrated positive effects in rodent models of Alzheimer's disease as well as Parkinson's disease (a degenerative disorder of dopaminergic neurons).^88, 89, 90^ Furthermore, psychostimulants themselves enhance AA levels and may exacerbate oxidative stress in the brain,^55, 56, 57, 85^ possibly increasing the levels of harmful AA metabolites. On the basis of our findings, GLP-1R signaling may oppose the effects of psychostimulants by affecting levels of AA as well as oxidative stress.

In this study, we provide biochemical and functional evidence supporting the notion that AA regulates DAT function by regulating DAT trafficking. We believe that the increase in DAT surface expression promoted by GLP-1R signaling represents a mechanism by which this neuropeptide controls both cocaine-induced increase in LS DA as well as cocaine actions. This AA-mediated mechanism is enhanced by peroxidation of AA leading to the formation of AA metabolites such as γ-KA. The treatment of hDAT cells with AA under conditions favoring lipid peroxidation significantly decreased DAT surface expression and transport capacity; the latter effect was also demonstrated with synthetic isoketals. These results are consistent with the known ability of isoketals to impair the function of plasma membrane proteins.^91^ Interestingly, AA alone was also able to significantly reduce DAT function. However, this reduction was smaller than that obtained under conditions favoring lipid peroxidation or in the presence of isoketals. These results provide a mechanism for the results reported by Chen *et al.*^52^ and Zhang and Reith,^53^ wherein the activity of DAT expressed in heterologous systems was reduced by AA.^52, 53^ They also mechanistically support the notion that GLP-1R signaling regulates DA homeostasis in the LS by decreasing AA levels. How exactly AA and its metabolites control DAT function and trafficking remains elusive. However, we hypothesize that covalent interactions of AA metabolites with specific lysine residues on the DAT support these actions.^59^ Notably, we were able to demonstrate a physical interaction between γ-KA and DAT by immunoprecipitation.

In summary, we have shown that GLP-1R agonism increases DAT function and membrane expression, decreases cocaine-induced increases in extracellular DA, and reduces endocannabinoid and AA signaling in the LS. This decrease is important as cocaine has been shown to increase 2-AG tone in the brain.^92^ This suggests that other brain centers targeted by cocaine and expressing the GLP-1R, albeit at lower levels, may exhibit a similar mechanism of reward modulation. These brain regions include the nucleus accumbens and VTA, which have been identified as targets of Ex-4 actions on brain reward.^11, 12, 13, 93, 94^ Nevertheless, this mechanism has still to be validated outside the LS. Our study points to septal GLP-1R as a potential novel target for the treatment of drug abuse. Targeting GLP-1R signaling represents an enormous translational opportunity as GLP-1 analogs are already available for clinical use.
