# Supplementary material for: Glucagon-like peptide 1 receptor activation regulates cocaine actions and dopamine homeostasis in the lateral septum by decreasing arachidonic acid levels
Source: Transl Psychiatry. 2016 May 17;6(5):e809–. doi: 10.1038/tp.2016.86 (PMC5070047; doi:10.1038/tp.2016.86)
Supplement: Supplementary Information [file tp201686x1.pdf]

Supplementary Information

Supplementary Figures:

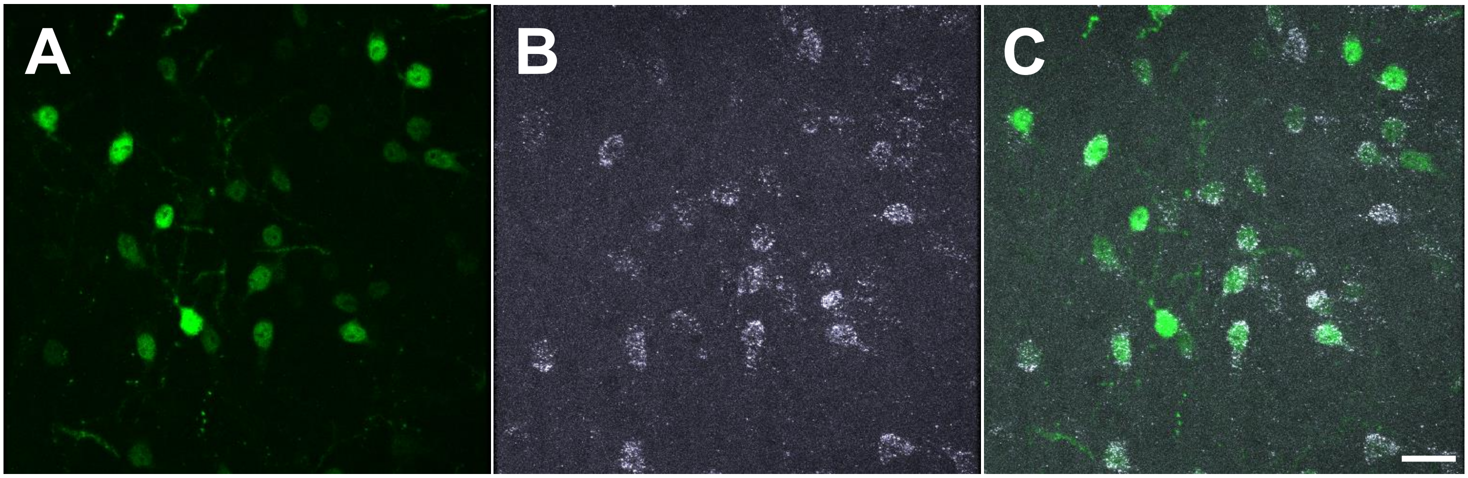

Supplementary Figure 1. *GLP-1R mRNA and mApple reporter are highly co-expressed in the LS of GLP-1R BAC reporter mice.* Mice expressing mApple protein under the GLP-1R promoter (A, green) express GLP-1R mRNA (B, white) mApple in the same cell bodies (C, merge). Confocal images of a z stack through 23 planes of view taken with a 40X objective. The scale bar in C represents 25 μm.

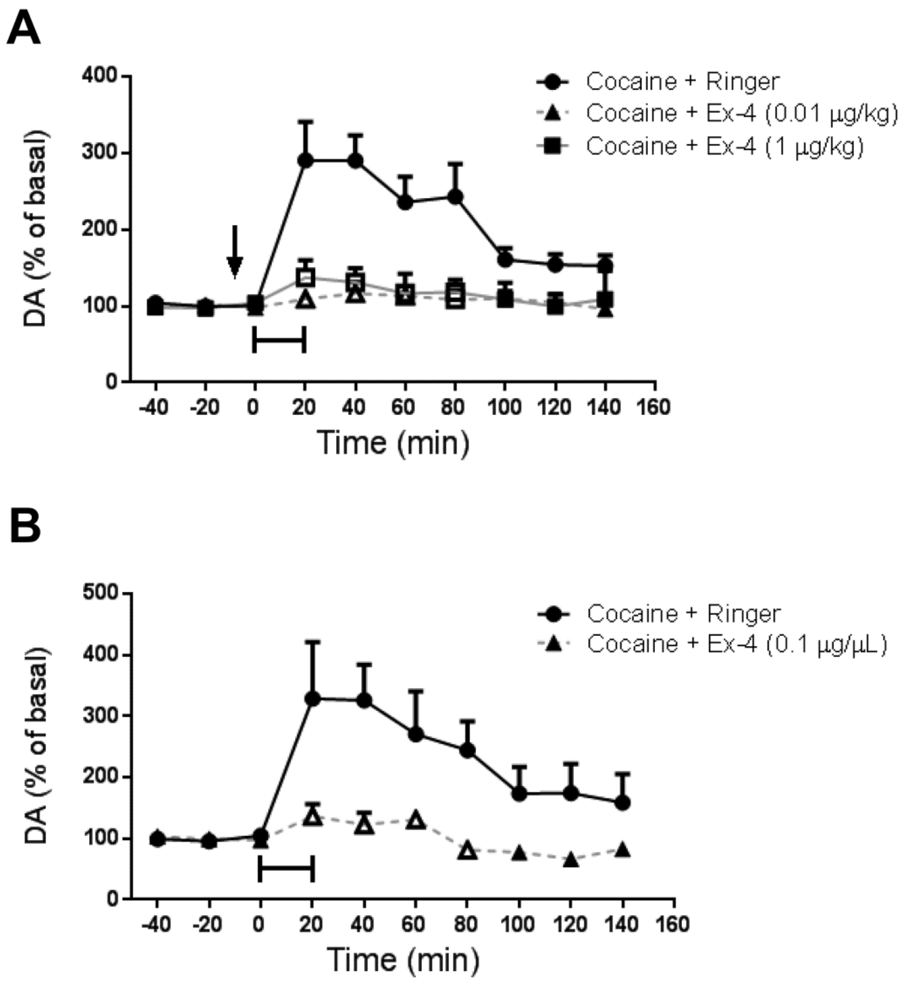

Supplementary Figure 2. *Extracellular concentrations of DA measured by microdialysis in the LS in anesthetized rats following systemic or local septal application of vehicle or Ex-4 concurrent with local cocaine perfusion.* (**A**) Effect of Ex4 0.01ug/kg i.p (solid triangles), Ex4 1ug/kg i.p. (solid squares), and cocaine alone (solid circles, 50µM, 0.9µl/min in 20min). Extracellular concentrations of DA are expressed as the percentage of basal levels in three fractions collected before the intervention. Significant two-way ANOVA. Open symbols indicate significance by *post hoc* test ($p<0.001$ for all open symbols compared to cocaine + ringer, n=4). (**B**) Effect of Ex4 0.01µg/µL (solid triangles), cocaine 50µM alone (solid circles). Extracellular concentrations of DA are expressed as the percentage of basal levels in three fractions collected before the intervention. Significant two-way ANOVA. Open symbols indicate significance by *post hoc* test ($p<0.001$ at time 20 and 40, $p<0.05$ at time 60, and $p<0.01$ at time 80, n=4-5).

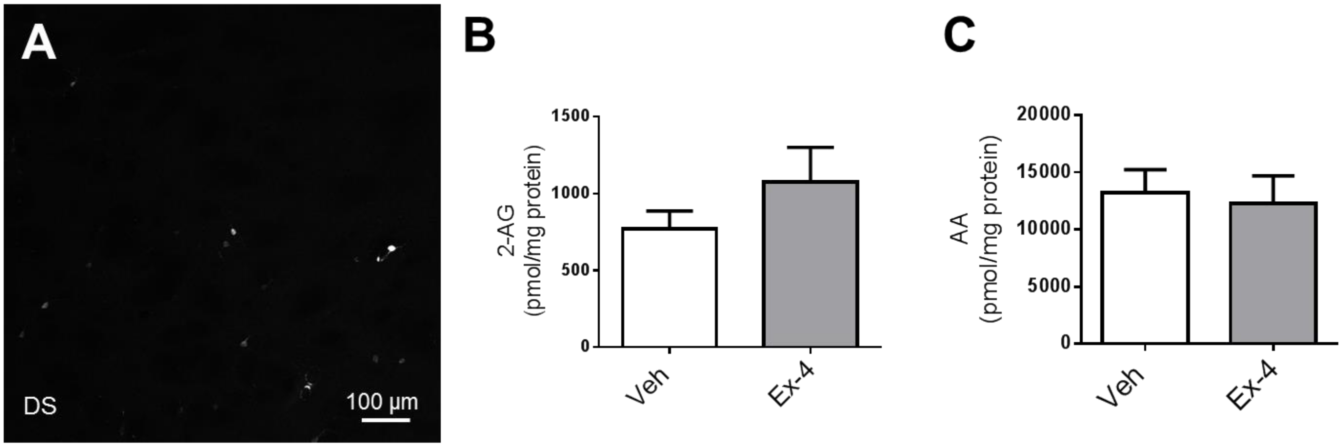

Supplementary Figure 3. *Ex-4 does not alter levels of 2-AG or AA in the dorsal striatum.* (**A**) Cells expressing the GLP-1R (white) are found at low levels in the dorsal striatum (DS), while the DAT is highly expressed (not shown). Confocal images of z stack through 13 planes taken with a 10X objective. (**B**) Ex-4 (2.4µg/kg, i.p.) administered 30min prior to sacrifice does not alter 2-AG levels in the DS (t(12)=0.29; p=0.78 by Student's *t* test; n=5-9). (**C**) Ex-4 (2.4µg/kg, i.p.) administered 30min prior to sacrifice does not alter DS AA content (t(12)=1.33; p=0.21 by Student's *t* test; n=5-9).

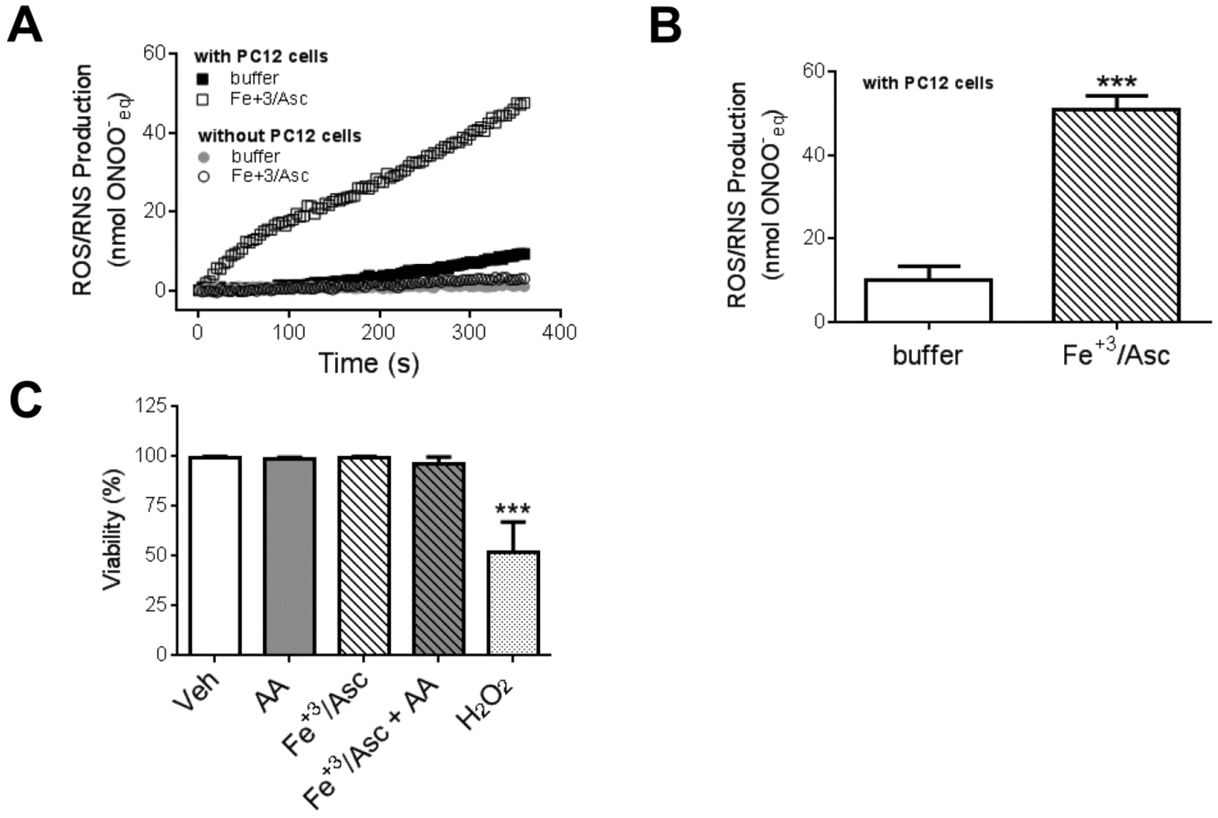

Supplementary Figure 4. *Fe^{+3}/Ascorbate solution induces reactive oxygen species/reactive nitrogen species formation in PC12 cells without changes in cell viability.* (**A**) Time course of reactive oxygen species/reactive nitrogen species (ROS/RNS) production in PC12 cells in absence (buffer, black squares) or presence of Fe^{+3}/Asc (open squares) at 37°C, evaluated with the fluorescent probe H_2DCFDA (2.5μM). Control experiments without cells in absence (buffer: gray circles) or presence of Fe^{+3}/Asc (open circles) were also performed. (**B**) Data are expressed as ROS/RNS production (in nmol of $ONOO^-$) in the first six minutes of incubation with the Fe^{+3}/Asc solution. Each value represents the mean ±S.E.M. of at least three different experiments. *** indicates significant differences between KH buffer and Fe^{+3}/Asc conditions ($t(5)=8.49$; ***$p=0.0004$ by Student's *t* test; n=5-9). (**C**) Cell viability of PC12 cells treated with AA (40μM) for 1 hour at 37 °C in absence or presence of Fe^{+3}/Asc. As positive control of cellular death cells were incubated with 2.2mM H_2O_2 (1h at 37°C). Each value represents the mean ±S.E.M. of at least three independent experiments.*** indicates significant difference (***$p < 0.0001$) with respect to control condition (Veh: 0.1% ethanol in KH buffer). ANOVA followed by *post hoc* test.

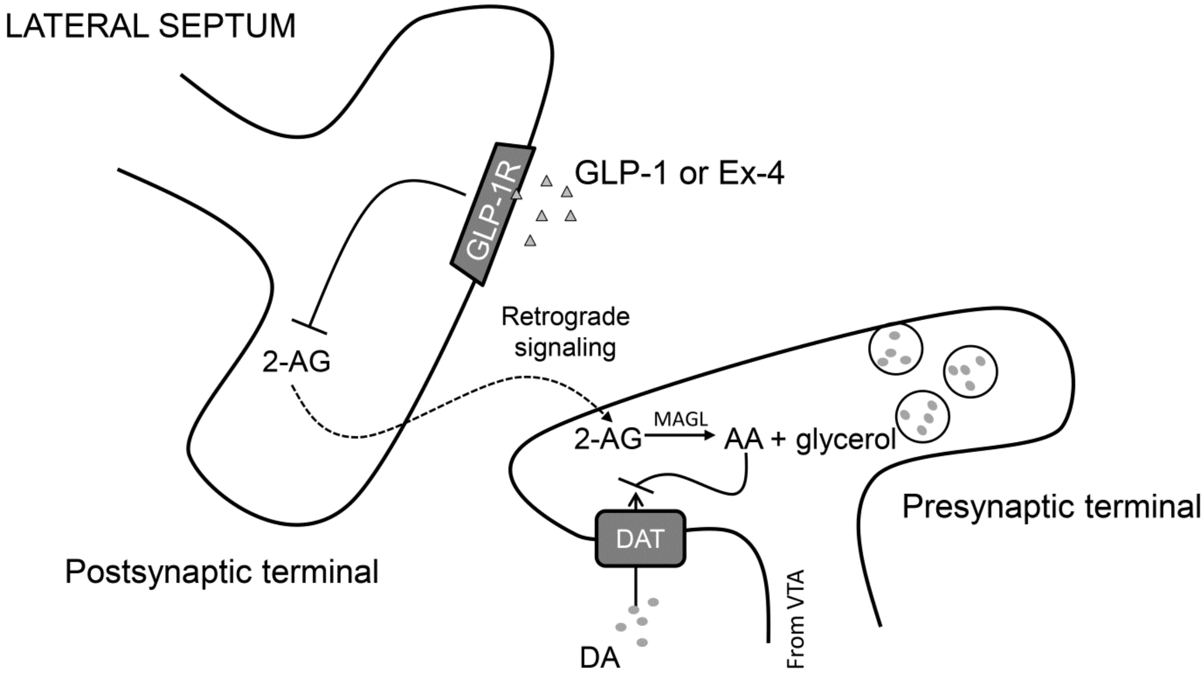

Supplementary Fig. 5. *Schematic depicting model for GLP-1R-mediated retrograde regulation of DA uptake.* MAGL = monoacylglycerol lipase.

Supplemental Materials and Methods:

Drugs and Materials. For *in vivo* studies Ex-4 was purchased either from Tocris Bioscience, UK or from Prospec, Israel. DA used as standards for HPLC was obtained from Sigma (St. Louis, MO, USA). Acetonitrile was chromatography grade (Merck, Darmstadt, Germany). All other chemicals used for microdialysis/HPLC (Merck or Fluka Chemie AG, Buchs, Switzerland) were of analytical grade or better and were used as supplied. GLP-1(7-36)-amide was purchased from Phoenix Pharmaceuticals (Burlingame, CA, USA) and Ex-9 was purchased from American Peptide Company (Sunnyvale, CA, USA) and was dissolved in artificial cerebrospinal fluid (aCSF) on the day of the experiment. Radiolabeled DA, [^{3}H]-DA (3.4-[7-^{3}H]dihydroxyphenylethylamine), was supplied by PerkinElmer Life Sciences (Waltham, MA, USA), and cold DA was obtained from Calbiochem (La Jolla, CA, USA). For *in vitro*

experiments, AA, goat anti-γ-ketoaldehyde adduct (neuroketals antibody, C-17, sc-130089), and peroxidase (HRP)-goat anti-rat IgG (sc-2006) were obtained from Santa Cruz Biotechnology (Santa Cruz, CA, USA). The rat anti-DAT (MAB369) antibody was obtained from Chemicon (Temecula, CA, USA). Bicinchoninic acid, sulfo-NHS-LC-LC-biotin, NeutrAvidin®-Agarose resin, HRP-rabbit anti-goat IgG (31402) and HRP-goat anti-rabbit IgG (31460) were obtained from Pierce (Rockford, IL, USA). Protein A and protein G Sepharose beads were obtained from GE Healthcare (Little Chalfont, UK). Lipofectamine® 2000 and 2.7-dihydrodichlorofluorescein diacetate (H_2DCFDA) were obtained from Invitrogen (Carlsbad, CA, USA). Salicylamine was obtained from Matrix Scientific (Columbia, SC, USA). Anti-dsRed was obtained from Clontech Laboratories (632496, Mountain View, CA, USA). Synthetic γ-ketoaldehydes were generously provided by Dr. Sean Davies (Vanderbilt University, USA).[1] AA used in *ex vivo* experiments, and all other chemicals used in this study were obtained from Sigma-Aldrich Chemical Co (St. Louis, MO, USA).

Generation of BAC transgenic mice: BAC transgenic mice were created by the Vanderbilt Transgenic and Embryonic Stem Cell Core as previously described[2] using a BAC clone containing the *Glp1r* gene (34.6 kb), which was flanked by 6 exons of the Dnahc8 gene (27 kb) and the first two exons of the Umod1 gene (18 kb) at the 5' and 3' ends, respectively; this clone was transferred into SW105 cells. An FRT-flanked antibiotic (Kan/Neo) cassette, containing a DNA fragment of an mApple fluorescent reporter was electroporated into the normal ATG start codon of the *Glp1r* gene within the BAC clone. Antibiotic-resistant colonies with the correct insertion were selected, and the FRT-flanked cassettes were removed via bacterial FLP recombination. The final BAC vector construction was confirmed by sequencing of all recombination junctions and by pulsed-field and standard fingerprint gels to correspond with predicted restriction digests. Validated vectors were injected into B6D2 embryos via pronuclear DNA microinjection. Embryos were injected into pseudopregnant B6D2 F1 hybrid females

(B6D2F1/J; Jackson Laboratories, Bar Harbor, ME). Genotypes were determined via PCR and fidelity of transgene expression in the CNS was determined by combined immunohistochemistry and ISH.

Combined immunohistochemistry and ISH *validation of GLP-1R BAC transgenic mouse.* To eliminate the possibility of aberrant mApple expression in our GLP-1R BAC transgenic mouse, we tested for co-localization of the mApple protein with GLP-1R mRNA. To accomplish this, we obtained brain sections from these mice from cortex through midbrain. Mice were deeply anesthetized with Nembutal sodium (120mg/kg; Akorn, Inc., Lake Forest, IL, USA) and transcardially perfused with RNAse-free PBS followed by RNAse-free paraformaldehyde (PFA). Brains were extracted, post-fixed for approximately 2h in 4% paraformaldehyde (PFA), cryoprotected overnight in 30% sucrose, and sectioned by microtome (60µm). Sections were preserved in sucrose at -20°C until use. Selected sections were then processed through an IHC assay (RNAscope kit) followed by immunohistochemistry. All probes, protocols, and RNAscope kit were generated by and obtained from Advanced Cell Diagnostics (Hayward, CA, USA). Following the RNAscope assay, sections underwent immunohistochemisty. Sections were blocked for 15min in 0.1M PB containing 2% goat serum and 0.1% triton-X then incubated in 1% sodium borohydride in 0.1M PB for 30sec. Sections were washed and placed in primary antibody. Cells expressing mApple were identified with rabbit anti-dsRed (1:1000) and HRP-conjugated goat anti-rabbit (1:200, Perkin Elmer, NEF812001EA), and visualized by immunofluorescence with fluorescein-Cy3 amplification to produce green fluorescence (Perkin Elmer, NEL704A001K). Sections were imaged with a confocal microscope (Zeiss LSM 510 Meta).

In vivo microdialysis in rats. Rats were placed in a stereotaxic instrument under servoflurane anesthesia (2%) in a mixture of 20% CO2 and 80% oxygen. The anesthesia was maintained during surgery and experiment. A small hole was drilled bilaterally to allow probes to be placed

into right and left LS. Probes (CMA/12, 2mm from CMA/Microdialysis AB, Stockholm, Sweden) were gently placed at the following coordinates in mm: AP:+1.0 ML:+ or- 0.4 and DV:-6.2 (relative to bregma). Rats were habituated 2h before the microdialysis sampling. Sampling (flow rate=0.9µl/min) was at 20min intervals for 60min (three fractions) prior to the intervention. After the seventh fraction was collected (10 total fractions), the rats were killed by rapid decapitation. The brain was extracted and sliced to verify the probe location. Only results from rats with probes verified in the LS are reported here. Ex-4 (local or i.p.) or cocaine (50µM local) were administered. Local Ex-4 was administered via a plastic-one cannula glue to the microdialysis probe, and cocaine was perfused via the 2mm microdialysis probe (0.9µl/min for 20min). All compounds were dissolved in Ringer.

Reactive oxygen/nitrogen species (ROS/RNS) measurements. In order to obtain the necessary conditions to induce γ-KAs formation from lipid peroxidation of AA, oxidative stress was promoted in hDAT cells. ROS/RNS formation in hDAT cells was measured using the fluorescence of H_2DCFDA according to previous reports from our laboratory.[3]

Supplemental References:

1. Amarnath V, Amarnath K, Matherson T, Davies SS, Roberts LJ. A Simplified Synthesis of Diastereomers of Levuglandin E2. *Synth Comm* 2005; **35**: 397–408.
2. Chen SX, Osipovich AB, Ustione A, Potter LA, Hipkens S, Gangula R, *et al.* Quantification of factors influencing fluorescent protein expression using RMCE to generate an allelic series in the ROSA26 locus in mice. *Dis Model Mech* 2011; **4**:537-547.
3. Egana LA, Cuevas RA, Baust TB, Parra LA, Leak RK, Hochendoner S*, et al*. Physical and functional interaction between the dopamine transporter and the synaptic vesicle protein synaptogyrin-3. *J Neurosci* 2009; **29**(14)**:** 4592-4604.
